# PATIENT PORTALS PROMOTE SHARED DECISION-MAKING AND ADOPTION OF HEALTHY HABITS RELATED TO MENOPAUSE

**DOI:** 10.1093/geroni/igae098.3399

**Published:** 2024-12-31

**Authors:** Roshni Singh, Fei Tang, Diana Ruiz, Stuti Dang

**Affiliations:** University of Miami, Miami, Florida, United States; Miami Veterans Affairs Healthcare System, Miami, Florida, United States; Miami Veterans Affairs Healthcare System, Miami, Florida, United States; Miami VA Healthcare System, Miami, Florida, United States

## Abstract

Perimenopausal women would benefit from a better understanding of menopause, and associated conditions and therapies. We implemented a menopause educational intervention called “MyHealtheVet to Enable And Negotiate for Shared decision-making” (MEANS) using the Veterans Affairs (VA) patient portal MyHealtheVet (MHV), in the Miami VA Healthcare System (MVAHS). Intervention participants received weekly secure messages with information on menopause. Here, we examine the effect of the MEANS intervention on SDM and adoption of healthy behaviors. We enrolled 269 women at MVAHS in MEANS. Participants’ average age was 53.2 years; 42.4% white, 43.1% black, and 24.2% Hispanic; 63.2% had a college education, and 95.9% already used MHV; 160 completed the six-month intervention and evaluation. Post-intervention, 57.5% reported that MEANS allowed them to get information that would otherwise be difficult to obtain, 79% said it increased their understanding of menopause, 80% felt more confident to discuss menopause treatments with their provider. Many reported discussing menopause-related conditions and treatments with their providers: 39% reported having discussed hormone therapy, 46% discussed osteoporosis, 33% discussed urinary incontinence. Many had adopted positive health behaviors: 8.1% had started taking hormones, 48% started calcium, 78% started Vitamin D; 38% started doing Kegel’s exercise; 69% began exercising, and 70% improved their diet. This pilot patient portal intervention shows that patient portals may offer a scalable tool to enhance SDM between patients and providers regarding menopause-related topics and to promote healthy behaviors among racially and ethnically diverse menopausal women. This strategy may be extrapolated to other non-urgent conditions.

